# Microbiome sharing between children, livestock and household surfaces in western Kenya

**DOI:** 10.1371/journal.pone.0171017

**Published:** 2017-02-02

**Authors:** Emily Mosites, Matt Sammons, Elkanah Otiang, Alexander Eng, Cecilia Noecker, Ohad Manor, Sarah Hilton, Samuel M. Thumbi, Clayton Onyango, Gemina Garland-Lewis, Douglas R. Call, M. Kariuki Njenga, Judith N. Wasserheit, Jennifer A. Zambriski, Judd L. Walson, Guy H. Palmer, Joel Montgomery, Elhanan Borenstein, Richard Omore, Peter M. Rabinowitz

**Affiliations:** 1 University of Washington, Department of Environmental and Occupational Health Sciences, Seattle, Washington, United States of America; 2 Washington State University, Paul G. Allen School for Global Animal Health, Pullman, Washington, United States of America; 3 Kenya Medical Research Institute, Centre for Global Health Research, Kisumu, Kenya; 4 University of Washington, Department of Genome Sciences, Seattle, Washington, United States of America; 5 University of Washington, Department of Global Health, Seattle, Washington, United States of America; 6 University of Washington, Department of Medicine, Seattle, Washington, United States of America; 7 University of Washington, Department of Epidemiology, Seattle, Washington, United States of America; 8 University of Washington, Department of Pediatrics, Seattle, Washington, United States of America; 9 Centers for Disease Control and Prevention, Division of Global Health Protection, Center for Global Health, Atlanta, Georgia, United States of America; 10 University of Washington, Department of Computer Science and Engineering, Seattle, Washington, United States of America; 11 Santa Fe Institute, Santa Fe, New Mexico, United States of America; 12 University of Washington, Department of Family Medicine, Seattle, Washington, United States America; Argonne National Laboratory, UNITED STATES

## Abstract

The gut microbiome community structure and development are associated with several health outcomes in young children. To determine the household influences of gut microbiome structure, we assessed microbial sharing within households in western Kenya by sequencing 16S rRNA libraries of fecal samples from children and cattle, cloacal swabs from chickens, and swabs of household surfaces. Among the 156 households studied, children within the same household significantly shared their gut microbiome with each other, although we did not find significant sharing of gut microbiome across host species or household surfaces. Higher gut microbiome diversity among children was associated with lower wealth status and involvement in livestock feeding chores. Although more research is necessary to identify further drivers of microbiota development, these results suggest that the household should be considered as a unit. Livestock activities, health and microbiome perturbations among an individual child may have implications for other children in the household.

## Introduction

The various taxa comprising the gut microbiome perform metabolic, signaling and immune functions in people and animals [1–3]. The maturation and structure of the gut microbiome can therefore have a long-term impact on health, and gut microbiome dysbiosis has been associated with various disease states, including malnutrition [4–10]. In order to promote health among young children, it is necessary to understand the environmental influences of gut microbiome development.

The maturation of the infant gut microbiome is marked by periods of abrupt change based on life events, dietary changes, and changes in environment [11]. Even after stabilizing following infancy, the gut microbiome of children still differs from that of adults, enriched with species which may support development [12]. Antibiotic use can have a major impact the constituents of the gut microbiome, which may increase a child’s susceptibility to pathogen colonization and invasion [13]. After reaching adulthood, the strains that exist in the gut microbiome are considered to be stable, potentially for decades [14].

In many community settings, people live in close contact with domesticated livestock and poultry. These animals, and their associated microbiomes, could influence the development and structure of human microbial communities through social interactions, animal husbandry activities, or indirectly through a shared environment [15–18]. However, studies assessing the overlap in the microbiomes of people and other animal species are rare. In the United States, a study by Song *et*. *al*. demonstrated that the skin microbiomes of people and their cohabiting dogs were significantly more similar than the skin microbiomes of unrelated humans and dogs. The study also found some evidence of gut microbiome sharing between the humans and cohabiting dogs [19]. Similarly, a study of the infant gut microbiome showed that the presence of pets in the household was associated with increased microbiome diversity [20]. To our knowledge, studies of microbiome overlap between humans, poultry, and cattle in low resource settings have not been conducted.

The built environment of human dwellings, including soil and surfaces, represents an additional possible driver for microbiome development, as microbiome has been found to differ across various environmental contexts [17]. For example, the most abundant microbes on the hands of Tanzanian women have been found to be soil-associated bacteria, whereas the most abundant bacteria on the hands of women in the US were found to be Staphylococcaceae and Propionibacteraceae [16]. However, whether the built environment influences the structure of the child gut microbiome in low resource settings is yet unknown.

Understanding the relationship between the rural household environment and microbiome development in children could lead to further insight into of the pathogenesis of dysbiosis-associated diseases and the design of household-level microbiome interventions to improve both child and animal health. To investigate the role of animal and household contact on gut microbiome of children in rural livestock-owning households, we studied the species composition of the gut microbiomes of young children, cattle, and chickens and of the microbiome of household surfaces in western Kenya. We evaluated a) whether children and their household animals and surfaces had similar microbiome constituents and b) what factors were associated with child gut microbial diversity.

## Materials and methods

### Study site

This study was conducted through a collaboration between the Kenya Medical Research Institute (KEMRI), Centers for Disease Control (CDC)-Kenya, Washington State University and the University of Washington. The study took place in western Kenya among villages participating in an established human-animal surveillance platform encompassing 1,800 mixed-crop and livestock agriculture households, with a total population of approximately 6,400 individuals [21]. Surveys of these households have shown that 90% own at least one species of livestock (cows, goats, or sheep) or poultry. Below, we use the word “livestock” to describe both cattle and chickens. The floors of living spaces and cooking areas are commonly packed soil. The household structures varied from grass thatched huts to brick buildings but in general were simple construction without insulation.

### Ethics approval and consent procedure

The study was approved by the human subjects review and animal care committees at the University of Washington as well as the KEMRI human subjects review board and the KEMRI Animal Care and Use Committee. Prior to study enrollment, community meetings were held to inform community leaders and members of the purpose of the study. Informed, written consent for participation in the study questionnaire and microbiome sampling, provided by the heads of household, was obtained in each person’s native language.

### Household selection

Eligible households were those with a child ≤5 years old, at least one cow, and at least one chicken, as of a demographic survey conducted in June 2014. From the approximately 250 households meeting these criteria, we randomly selected 180 for sampling. If a household had more than two children ≤5 years old, the children were ordered in a random list from which the first two were approached to enroll. If consent was refused or the household head was unreachable, the next random household was approached.

### Household demographics

Teams of community interviewers and veterinary technicians were trained in questionnaire delivery and microbiome sample collection. At each household to be sampled, a community interviewer conducted a brief survey with the head of the household to determine the children’s interaction with household livestock, exposure of children and livestock to antibiotics, and livestock care practices. The survey is included in the supplemental materials. Data on household demographics and wealth were collected from an ongoing surveillance study in the same area [21]. Household wealth status was calculated as an asset-based wealth score using Principal Components Analysis.

### Sample collection and transport

Sampling took place between October and November 2014. The child’s caregiver was provided with a stool collection container and was instructed to collect the child’s first stool the next morning. The following morning veterinary technicians arrived to collect the child’s stool sample, swabs of household surfaces (one swab of 10 cm^2^ of the floor of the cooking area [usually outside], and one swab of 10 cm^2^ of the floor of the living area [usually inside]), cattle fecal samples, and chicken cloacal swabs. Animal samples were collected in accordance with standard animal handling techniques (see Supplementary Methods, Sample Collection for details). All samples were labeled, sealed in individual containers, and transported on ice to the field laboratory in Lwak, Kenya where they were refrigerated at -20°C until the end of the day when they were transported on ice to the laboratory in Kisian, Kenya (approximately 1 hour transit time) where they were frozen at -80°C.

### DNA extraction

DNA was extracted from all samples in Kenya using the MoBio PowerFecal^®^ kit according to the manufacturer’s instructions (MoBio Laboratories Inc, Carlsbad, CA). Samples and DNA were handled under sterile conditions in a UV-treated laminar-flow biosafety hood. Extracted DNA was frozen at -80°C and then shipped on dry ice to Washington State University, Pullman WA, USA.

### Amplification and 16S-rRNA sequencing

Up to 10 ng of each sample was used as input to a two-step dual-indexing PCR targeting variable regions 1, 2, and 3 of the 16S-rRNA gene [22]. Primers sequences are included in the Supplemental Methods (S1 and S2 Tables). Individual libraries were pooled (10–12 libraries/pool), purified, and size selected with Ampure beads (Beckman Coulter). Purified pools were quantified using a Quant-IT High Sensitivity fluorescence assay (Invitrogen), normalized to 4 nM each, and 180 to 230 individually barcoded libraries were pooled for simultaneous sequencing on a MiSeq (Illumina, San Diego, CA) utilizing the v3 chemistries and a 2 x 300 bp paired-end cycle sequencing run.

### Microbiome data processing

Sequences were subjected to on-instrument quality control and raw sequence reads were de-multiplexed. Due to the length of the sequenced amplicon and lower reverse read sequence quality, forward and reverse reads were unable to be joined reliably and the analysis relied on the forward reads only [23]. We applied the sequence quality filtering approach recommended by Bokulich *et al*. [24] and also removed any samples with fewer than 1,000 reads following this filtering process. When technical replicates were present, we selected the replicate with the higher average base quality as input for Operational Taxonomic Unit (OTU) picking. We used *de novo* OTU picking for consistency with other studies and to retain reads from uncharacterized taxa for downstream analysis [25] (see Supplemental Methods for details). We assigned taxonomy to each OTU using UCLUST and aligned representative sequences for each OTU to the SILVA database using PyNAST [26, 27]. We filtered the following classes of OTUs from the dataset: singleton OTUs, OTUs whose representative sequences failed to align to the SILVA database with >65% identity, OTUs that were identified as chimeric using the uchime-ref algorithm implemented in the *vsearch* library [17], and OTUs that were only present in one sample [24]. Resulting OTU tables were additionally rarefied to a constant number of reads that preserved 80% of the samples, and subsequently OTUs present in only one sample were removed. These rarefied tables were used only for diversity and correlate analyses (see below), but the samples lost during rarefication due to insufficient numbers of reads were excluded from all analyses.

### Diversity indices and statistical analysis

We constructed a phylogenetic tree of all OTUs using FastTree [28]. The resulting tree was used to calculate the unweighted UniFrac distances between all samples [29]. To compare the taxonomic compositions of samples from different origins or hosts, we conducted a principal coordinate analysis (PCoA) on the UniFrac distances between all samples.

To address the primary question of whether children shared more microbes with hosts in their own households than with hosts in other households, we calculated pairwise distances between all samples using Bray-Curtis abundance metrics through QIIME [30, 31]. The distributions of OTU sharing measured within and between households were compared using the Wilcoxon rank-sum test with an α significance level of 0.05. For complete and near-complete household sample sets (i.e., household for which at least four sample types were available), we conducted an additional sharing analysis by calculating the total relative abundance of human microbiome OTUs that were also present in animals or surfaces sampled in the same household. We used these to create stacked bar plots showing the fraction of each human sample associated with OTUs that are also found in any other sample type in their household.

We explored the correlates of child gut microbiome diversity as measured by OTU count, using linear mixed models clustered by household. We tested univariable correlations using the predictors of child age and sex, household livestock ownership and asset-based wealth status, and reported child livestock caretaking practices (as reported through questionnaire in Supplemental Materials). We also evaluated correlates of the household-level OTU sharing metrics, measured by the Bray-Curtis distance measures, using linear regression. Predictors included household livestock ownership, household wealth status, and number of household members. Independent variables showing association with the diversity outcome at p<0.2 were added to a multivariable model.

### Sensitivity analyses

We conducted three assessments to determine whether the household OTU sharing results were sensitive to our choice of methods. First, we applied and compared three different OTU picking approaches: (i) 97% similarity closed reference OTU picking using QIIME 1.9.1 [31], (ii) 97% similarity *de novo* OTU picking using QIIME 1.9.1 [31], and (iii) Swarm v2 OTU clustering [32, 33]. Results obtained with the second OTU picking methods are described below and details on all methods are included in the Supplemental Methods. As an additional sensitivity analysis, we calculated two sharing metrics in addition to the Bray-Curtis metric: (i) the Jaccard binary distance metric and (ii) asymmetric sharing metrics (for each pair of samples, the fraction of OTUs in one sample that were also found in the other and the proportion of reads assigned to such shared OTUs). Finally, to examine whether antibiotics influenced sharing levels between household members and their environment, we repeated the primary sharing analysis excluding those samples for which caregivers reported antibiotic use among children, cattle, or chickens in the one-month period prior to sample collection.

## Results

### Study households and subject demographics

Among 180 households contacted, sampling was performed at 158, yielding 184 child stool samples, 158 cattle stool samples, 158 chicken cloacal swab samples, and 316 household surface samples. Table 1 shows the demographic data for households that had both questionnaire data and microbiome data for at least one child. These households reported owning an average of 12 chickens and five cattle. Study children were predominantly (60%) female and ranged in age from 10 months to 66 months at the time of sampling (some children were over age five because sampling was conducted after initial selection and consent for the study). Based on caregiver surveys, children were involved in several aspects of livestock caretaking, including herding (67%), feeding (64%), and cleaning livestock areas (59%). According to respondents, 35% of the children had received antibiotics in the past month, while 12% of sampled cattle and 6% of sampled chickens were reported to have received antibiotics. Chickens were reported to commonly enter human dwellings.

**Table 1:** Characteristics of sampled households, children, cattle, and poultry, western Kenya 2014 (n = 117 households).

Characteristic	Sample mean(SD) or n(%)
Household Characteristics	
Household buildings, number	2.1 (0.9)
Livestock ownership, number	
Cattle	5.1 (5.3)
Poultry	12.1 (8.0)
Sheep	1.9(3.4)
Goats	2.9 (3.3)
Child characteristics	
Age, months	39.7 (13.3)
Sex, % female	75 (60%)
Recent antibiotic use, yes/no	46 (35%)
Livestock activities, yes/no	
Feeding	83 (63%)
Milking	26 (20%)
Herding	82 (63%)
Slaughtering	23 (18%)
Caring for sick animals	1 (1%)
Taking to market	1 (1%)
Cleaning livestock areas	77 (59%)
Sampled cattle characteristics	
Age, months	46.9 (16.2)
Recent antibiotic use	14 (12%)
Enters the house	22 (19%)
Sampled poultry characteristics	
Recent antibiotics	7 (6%)
Enters the house	1129 (98%)

### Microbiome sequencing and quality

Initial sequencing produced 579 samples with complete metadata with a total of 34,975,832 reads. 409 samples passed rarefication thresholds after OTU picking. This set of 409 samples included 32,228,377 reads in total, an average of 78798 ± 100041 reads per sample, with mean R1 and R2 base qualities of 31 ± 1.7 and 26 ± 1 respectively (Table 2). Trimming and quality filtering reduced the total read count to 21,187,109.

**Table 2:** Sequencing data quality and *de novo* Operational Taxonomic Unit (OTU) count results, by sample type.

	Read counts,	Base Quality		97% *de novo* OTU counts (% unassigned)			
		R1	R2	Unrarefied		Rarefied	
Sample type (n)	Mean±SD	Mean±SD	Mean±SD	Mean±SD	Total	Mean±SD	Total
Chicken (36)	54468±58025.2	31±2.2	26±1.3	1896±1168.1 (49±25.28%)	27370 (37%)	749±429.2 (50±25.87%)	11936 (36%)
Cooking area (69)	72895±72964.4	31±1.7	27±1.2	5314±4962 (15.5±6.79%)	135681 (20%)	1625±635.2 (14.2±6.67%)	42489 (18%)
Cow (125)	75793±71498.5	31±1.2	27±0.5	8654±4904.4 (20.9±5%)	186838 (25%)	2732±590 (19.5±4.79%)	63858 (23%)
Human (143)	89517±139183.1	31±1.1	27±0.5	5250±4394.6 (2.1±1.43%)	168876 (3%)	1400±368.6 (2.2±1.48%)	47772 (3%)
Living Space (34)	81873±64716.8	29±2.6	26±1.7	4790±2343 (12.7±5.02%)	76464 (17%)	1756±674.1 (11.1±4.59%)	29210 (15%)
Total (409)	78798±100041	31±1.7	26±1	6025±4784.5 (14.8±15.22%)	457322 (18%)	1835±835.6 (14.2±15.28%)	148783 (18%)

This final analytic sample set included 156 total households that had microbiome data for at least one sample, providing data for 143 children, 125 cows, 36 chickens, 34 living spaces, and 69 cooking areas. In 100 households there was at least one child sample and one cow sample available for comparison, while 22 households had more than one child available for comparison. Due to the low availability of chicken and living space data that remained after processing, only one household included a full set of analytic data of all five sample types.

### Identified OTUs

We initially identified 1,350,823 OTUs with 97% *de novo* clustering. This set was reduced to 580,645 OTUs after singleton removal, 479,381 OTUs after chimera filtering, and 464,847 OTUs after removal of sequences that failed to align to the database. After rarefying samples to 7,109 reads and filtering again single-sample OTUs, 2,828,844 reads, 406 samples and 151,296 OTUs remained. The average number of OTUs identified varied by sample type after rarefication, with human samples having the fewest OTUs on average (Table 2). Samples from cows displayed the highest α diversity, with an average of 2,700 observed OTUs per sample. However, human samples had the highest proportion of OTUs that could be assigned a taxonomic classification.

**Fig 1:**
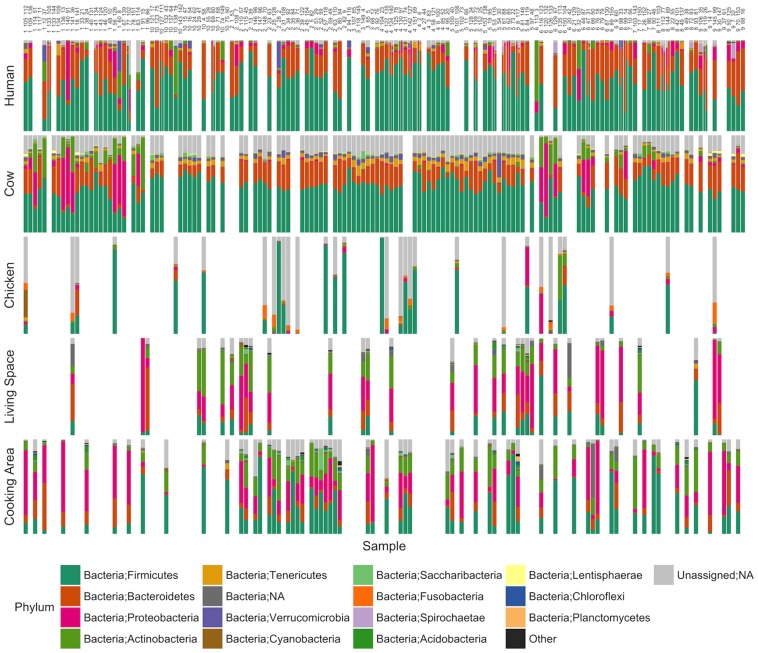
Bar charts representing the relative abundance of phyla in the gut microbiomes of children, cattle, and chickens, and in environmental microbiomes from living spaces and cooking areas in households in western Kenya. Each column represents a single household. In households where two children were sampled the human bar plot is divided evenly down the center. Samples with missing data are blank while unassigned taxa are gray.

**Fig 2:**
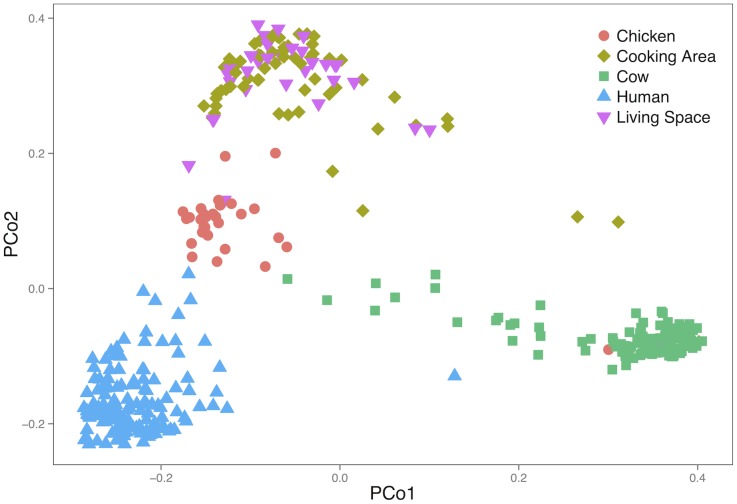
Principal coordinate analysis of unweighted UniFrac distances representing phylogenetic clustering of gut and environmental microbiome constituents in samples from households in western Kenya.

The most abundant phyla among all samples were Firmicutes and Bacteroidetes, followed by Proteobacteria (56.4%, 27.7%, and 5.1% average abundance, respectively, Fig 1). OTUs assigned to the family Prevotellaceae were found at an average abundance of 20.1% in human samples. Cow samples were largely dominated by Firmicutes and Bacteroidetes, although a smaller subset of cow samples (13 out of 123) were dominated instead by Proteobacteria and Actinobacteria OTUs. Chicken samples featured the highest proportion of reads (38.9%) belonging to OTUs with unassigned taxonomy, including 6 out of 31 samples with greater than 80% of reads from OTUs of unknown taxonomy. The taxonomic groups present in the environmental samples were highly variable, with Proteobacteria as the most abundant (35.6%) in living space samples and Firmicutes in cooking area samples (28.1%). A PCoA plot demonstrated that overall the community structures within each sample type were similar to each other and relatively distinct from community structures in other sample types (Fig 2).

### Microbial sharing within and between households

**Fig 3:**
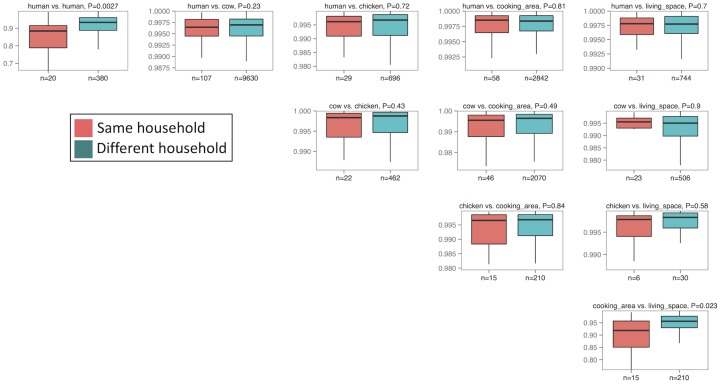
Pairwise comparisons of the distribution of Bray-Curtis abundance distance metrics comparing samples within and between households in western Kenya. Children within the same household and surfaces within the same household show significantly similar microbial communities.

Using the Bray-Curtis metric to assess the similarity between samples demonstrated a general trend by which members or household surfaces from the same household had higher OTU sharing compared to members or household surfaces from different households, although most comparisons were not statistically significant (Fig 3). Similarly, the gut microbiome of children showed a trend (which again, was not statistically significant) toward sharing with the microbiome of cows in the same household. There was also no significant overlap between child gut microbiome and the microbiome of chickens, or surfaces within their own household compared to those of other households. Examining the similarity of the gut microbiome of children living in the same household (across 20 households that included two children) demonstrated that sharing between the gut microbiome of children from the same household was significantly higher than sharing between children living in different households (p = 0.0027; Bray-Curtis distance, Wilcoxon rank-sum test; Fig 3). The surface microbiomes of the cooking area also exhibited more overlap with living spaces in the same household than with other households (p = 0.023; Bray-Curtis distance, Wilcoxon rank-sum test; Fig 3). These results did not qualitatively differ when using Jaccard binary distance (S1 Fig), asymmetric proportions of shared OTUs (S2 Fig), or when using other OTU picking methods (S3 and S4 Figs).

**Fig 4:**
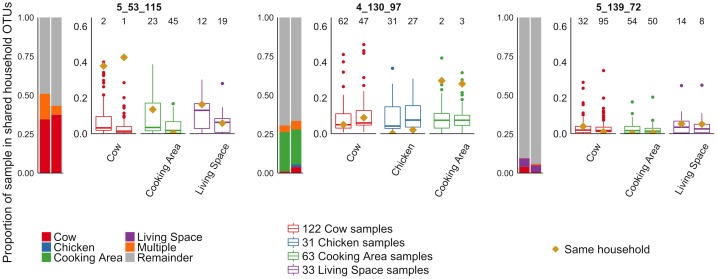
Proportion of microbiome sharing between children and other household samples in three households in western Kenya. Each household represented here had two children, represented by the vertical bar and box plots. The bar graphs on the right side of each panel illustrate the proportion of microbes shared between the child and their cow, chicken, cooking area, or living space. The box plots in each panel show the distribution of microbiome sharing between the child and samples from other households, compared to the proportion shared with their own household sample, represented by a gold diamond. The number on top of each box plot represents the rank of the sharing within the same household among sharing values calculated with all other households (e.g., “1” indicates a case where the sharing with the same household was the highest compared to sharing with any other households). The first panel shows a household in which both children had a high level of microbiome sharing with the cow in their household compared to cows in other households. The second panel shows a household in which both children had a high level of sharing of their gut microbiome with the surface in the cooking area of their household. The final panel shows a household in which the children did not show sharing with any of their household samples.

Fig 4 illustrates the proportion of the OTUs in each child’s gut microbiome shared with livestock or surfaces within the same household for three different households. In some households, children demonstrated notably higher proportions of microbes shared with cattle, chickens, or surfaces of their own household compared to other households. In other households this sharing was not observed (and such sharing was not significant on average), suggesting unknown variables may impact whether substantial microbe sharing occurs between children and cattle in a given household. A figure illustrating the proportion of sharing in all households that had data from at least one child and two additional sample types is included in the Supplemental Results (S5 Fig).

When we repeated the primary sharing analysis among the subset of children and livestock that did not report antibiotic use in the prior month, we found similar results (S6 Fig). However, since only ten households included two children for whom caregivers reported no antibiotic use, this effect was no longer statistically significant.

### Correlates of microbiome diversity and sharing

In univariable analysis, child age (in months) and a caretaker report that the child had ever fed household livestock were positively associated with higher OTU count (β = 6 OTUs, 95% CI: 1, 11 OTUs; β = 178 OTUs, 95% CI: 45, 310 OTUs, respectively). Household wealth score was significantly negatively associated with OTU count (-47 OTUs, 95% CI: -87, -6 OTUs). In multivariable analysis, household wealth status and child livestock feeding remained statistically significant in their association with gut microbiome diversity (S3 Table).

Using the Bray-Curtis divergence metric as the outcome, none of the tested factors (including number of animals owned, number of household members, household wealth, child age child sex, reported child interactions with livestock, or livestock housing) showed univariable associations with microbiome similarity between children and cattle. Although microbial similarity was low between humans and chickens overall (Fig 3), the gut microbial communities of boys had significantly more similarity to chickens within their household than those of girls (β = 0.01, 95% CI 0.003, 0.02). A higher number of latrines available to the household showed borderline association with lower microbiome similarity among the children in a household (S3 Table). Total household livestock count and both children receiving antibiotics in the past month were associated with closer microbiome similarity between two children. Due to small numbers of comparisons, we did not perform multivariable analysis on the sharing metrics between children. Results did not differ when using the Jaccard distance metric as the outcome (data not shown).

## Discussion

In this study, we describe the microbial constituents of the gut from young children in western Kenya, the gut of their household livestock, and their household environment. Overall, we found that the gut microbiome of children is more likely to be shared with other children within the household compared to children from different households. Overall, we did not observe significantly greater microbiome sharing between children and livestock or surfaces in the same households than between households. Further research will be necessary to determine the characteristics of households that show higher microbial overlap between host species.

The level of microbiome sharing observed between children in the same household was associated with reported antibiotic use among the children. However, removing those children who used antibiotics from the analysis did not meaningfully change the sharing effect size between children in the same household. There are several possible reasons that this effect was observed. First, the microbiome perturbation could lead to increased microbe sharing in the recolonization process. Alternatively, antibiotics could eliminate particular microbes, leaving common core constituents between individuals. Our finding that the dominant gut microbiome phyla in children sampled were the Firmicutes and the Bacteriodetes, followed by Proteobacteria is consistent with other studies [34–38]. In our sample, factors associated with increased child gut microbiome diversity included lower household wealth, greater child age, and whether the child was reported to help feed livestock. Among young children, age has consistently been associated with increasing microbiome diversity [39, 40]. Our finding that higher wealth status was associated with lower gut microbiome diversity was consistent with a recent study in Malaysia reporting that the children in the group of the highest socio-economic position had the lowest gut microbiome diversity [41]. Higher socioeconomic status could result in higher levels of hygiene, leading to lower gut microbiome diversity. Ostensibly, higher wealth index could also suggest higher access to antibiotics for children in rural areas, which could decrease microbial diversity. However, this cohort was enrolled in a long-term surveillance study that provided free access to healthcare, and reported antibiotic use was inversely correlated with household wealth index among these children. Finally, those children who engaged in livestock feeding may have more interactions with animal microbiomes through direct contact with animals and manure, which could increase their microbiome diversity. These exploratory findings should be investigated in future studies.

In the analysis reported above, we tested the sensitivity of our results based on three different OTU picking methods. While each clustering method has strengths and weaknesses, for microbiomes that contain high proportions of sequences not represented in databases, *de novo* picking is preferred to closed-reference picking [25]. Only 28.5% of reads in this study were mapped to the Silva database (15.6% more than were assigned using the Greengenes database). The *de novo* and closed reference picking methods showed very similar results, while the Swarm picking method led to lower OTU counts and the resulting analyses showed no statistically significant microbiome sharing between any household samples. This may be because, for this dataset, the Swarm method created a large number of OTUs with singleton reads, which were subsequently filtered out. For complex, diverse microbiome datasets across different host species, such as in this case, *de novo* picking may be the most appropriate option [25]. Regardless of the picking method used, chicken microbiome samples showed a low proportion of OTUs that could be assigned, indicating the need for improved reference libraries for non-human host species.

This study was conducted in a unique population of livestock-owning households in western Kenya. Strengths of this study include the relatively large sample size and a representative sampling frame. Nonetheless, the study also had a number of limitations. The cross-sectional nature of the sampling limits any inference regarding the directionality of transmission of microbiome elements between people, animals, and surfaces. Additionally, nearly 48% of the samples (78% of living space, 77% of chicken, 55% of cooking area, 19% of cow, and 13% of human samples) either failed PCR amplification or did not produce usable libraries during sequencing. This low yield may be due to low DNA mass or the presence of PCR inhibitors. In particular, the cloacal swabs from the chickens often did not produce sufficient DNA for analysis. Previous studies of chicken gut microbiomes have involved harvesting of entire gut contents, which provides large quantities of DNA [42, 43], while our study relied on cloacal swabs from living animals. Additionally, although samples were immediately placed on ice, transit time to the facility where they were frozen was approximately one hour. This may have led to some DNA degradation, although previous studies have found that room temperature storage for up to three days did not impact DNA quality[44]. It is possible that physical factors such as type of household construction and duration of stay in household could affect microbiome composition and sharing- while our survey did not include information about construction type this should be explored in future studies. Finally, the questionnaire-based predictors for the analysis of correlates of microbiome diversity and sharing involved some subjectivity and were subject to the memory of caregivers. We anticipate that this would lead to a nondifferential, nullifying impact on the observed relationships.

## Conclusion

In rural villages, the gut microbiome of children appears to be shared between children within the same household. Some children may also share microbiome components with nearby animals, although this effect does not appear to be consistent across the population. Microbiome diversity of children varied with wealth status and livestock contact. Because the gut microbiome plays a critical role in child health, our findings suggest that the health of household members should be considered linked, and that antibiotic use and livestock interactions among a single child in a household can have implications for the microbiome of other children in the home.

## Supporting information

S1 Fig Pairwise comparisons of the distribution of Binary Jaccard distance metrics comparing samples within and between households in western Kenya. Lower values represent closer OTU overlap.
(TIF)

S2 Fig Pairwise comparisons of the distribution of Asymmetric distance metrics comparing samples within and between households in western Kenya. Lower values represent closer OTU overlap.
(TIF)

S3 Fig Pairwise comparisons of the distribution of Bray-Curtis abundance distance metrics using closed reference picking comparing samples within and between households in western Kenya. (TIF)

S4 Fig Pairwise comparisons of the distribution of Bray-Curtis abundance distance metrics using Swam picking comparing samples within and between households in western Kenya. (TIF)

S5 Fig Proportion of microbiome sharing between children and other household samples in three households in western Kenya. Each household is represented by on panel including the vertical bar and box plots. The bar graphs on the right side of each panel show the proportion of microbes shared between the child and their cow, chicken, cooking area, or living space. The box plots in each panel show the distribution of microbiome sharing comparing the child to samples from other households, compared to the proportion shared with their own household sample, represented by a gold diamond.
(TIF)

S6 Fig Pairwise comparisons of the distribution of Bray-Curtis abundance distance metrics comparing samples within and between households in western Kenya excluding children whose caregivers reported that they received antibiotics in the prior one month. (TIF)

S1 Table Primer designs for PCR steps 1 and 2. (DOCX)

S2 Table Master primer table with exact primer sequences. (DOCX)

S3 Table Excel file of sample-level microbiome data. (CSV)

S4 Table Correlates of individual-level diversity (rarefied OTU count), multivariable linear mixed regression (N = 107). (DOCX)

S5 Table Correlates of microbial similarity (Bray-Curtis distance metric) between children (N = 22). (DOCX)

S1 File Supplementary Materials, including methods, results, and references. (DOCX)
