# Supplementary figures and images for: Microbiome sharing between children, livestock and household surfaces in western Kenya
Source: PLoS One. 2017 Feb 2;12(2):e0171017. doi: 10.1371/journal.pone.0171017 (PMC5289499; doi:10.1371/journal.pone.0171017)

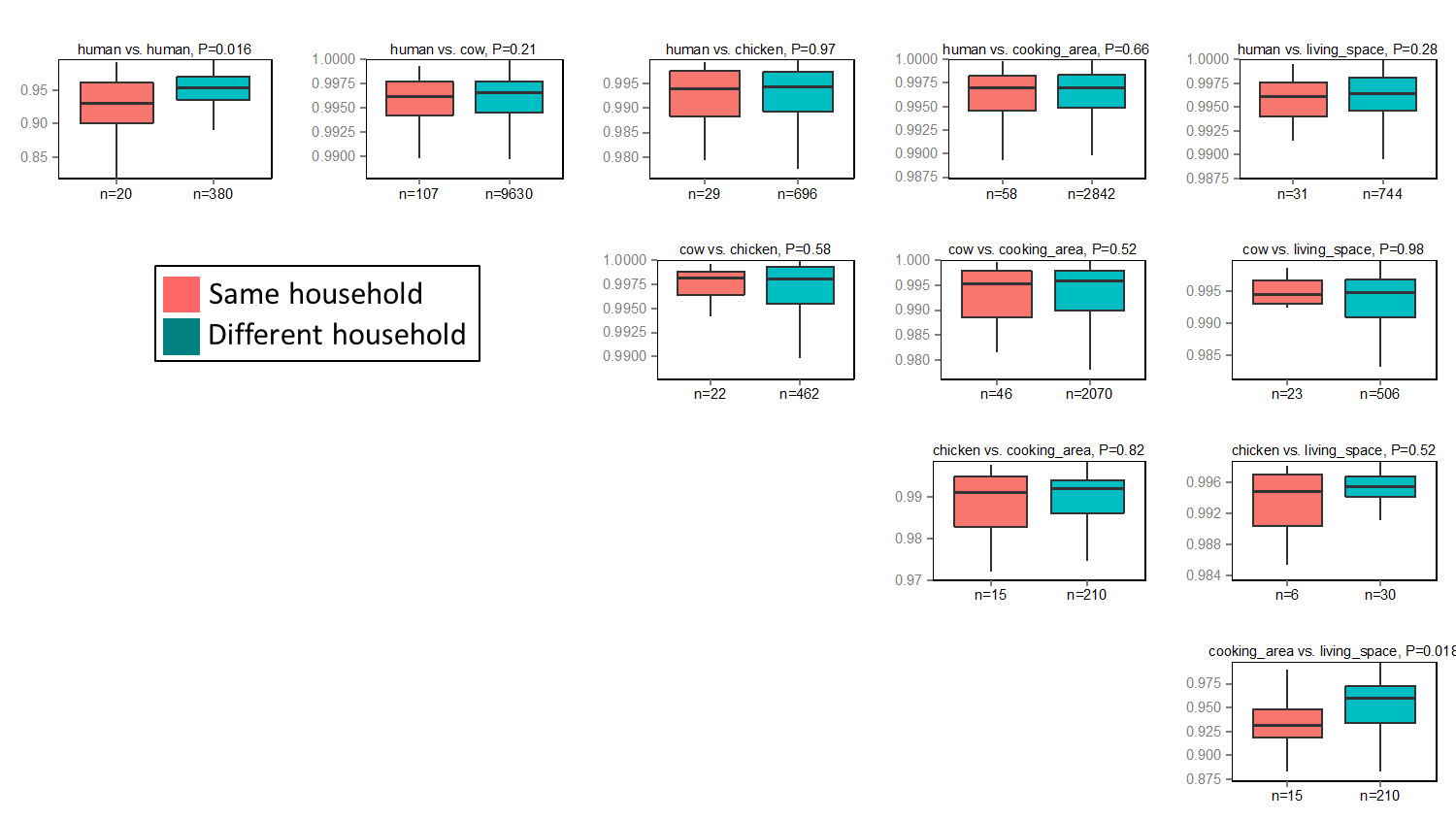

Supplement: S1 Fig — Lower values represent closer OTU overlap. (TIF) [file pone.0171017.s001.tif]

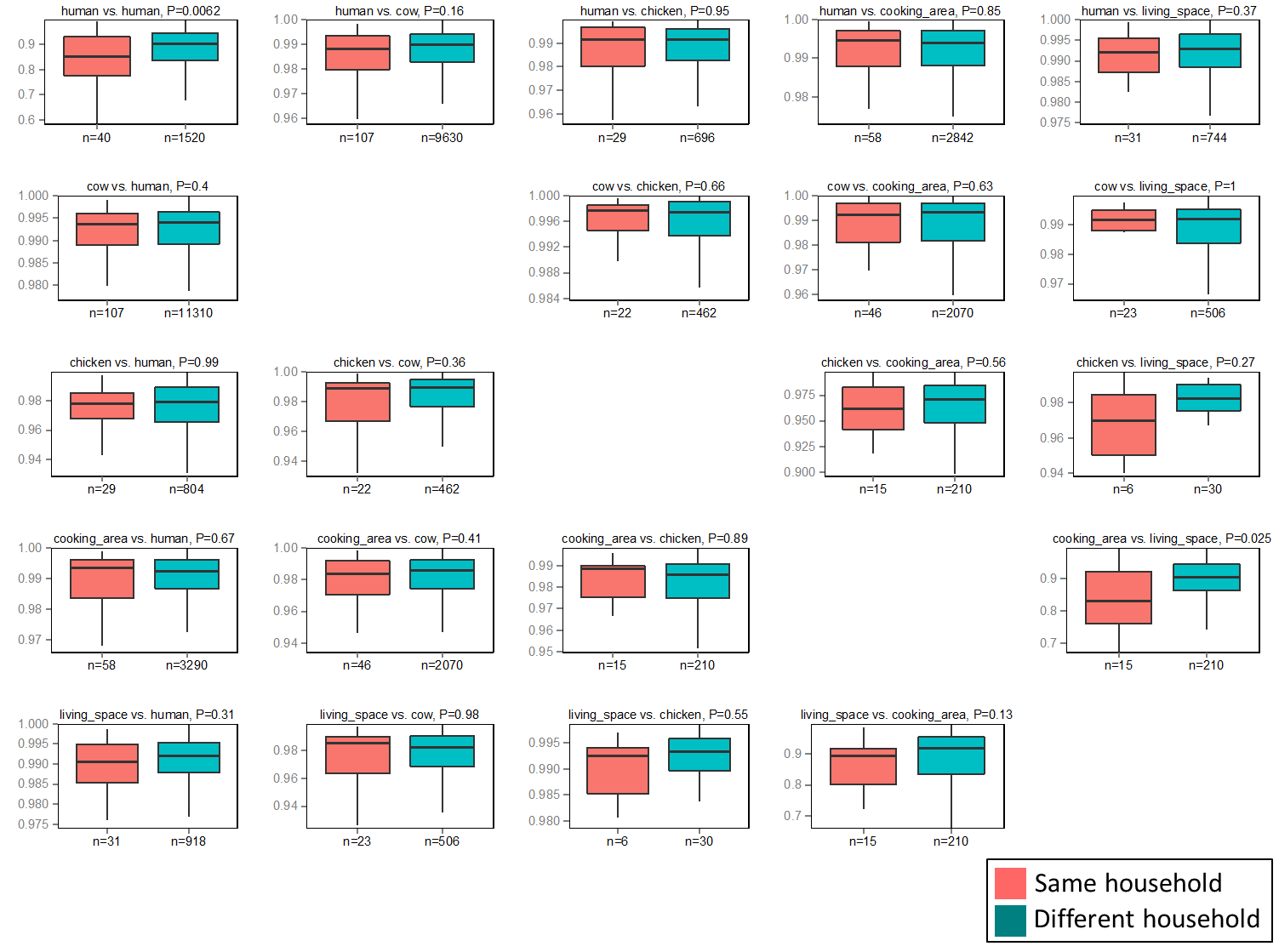

Supplement: S2 Fig — Lower values represent closer OTU overlap. (TIF) [file pone.0171017.s002.tif]

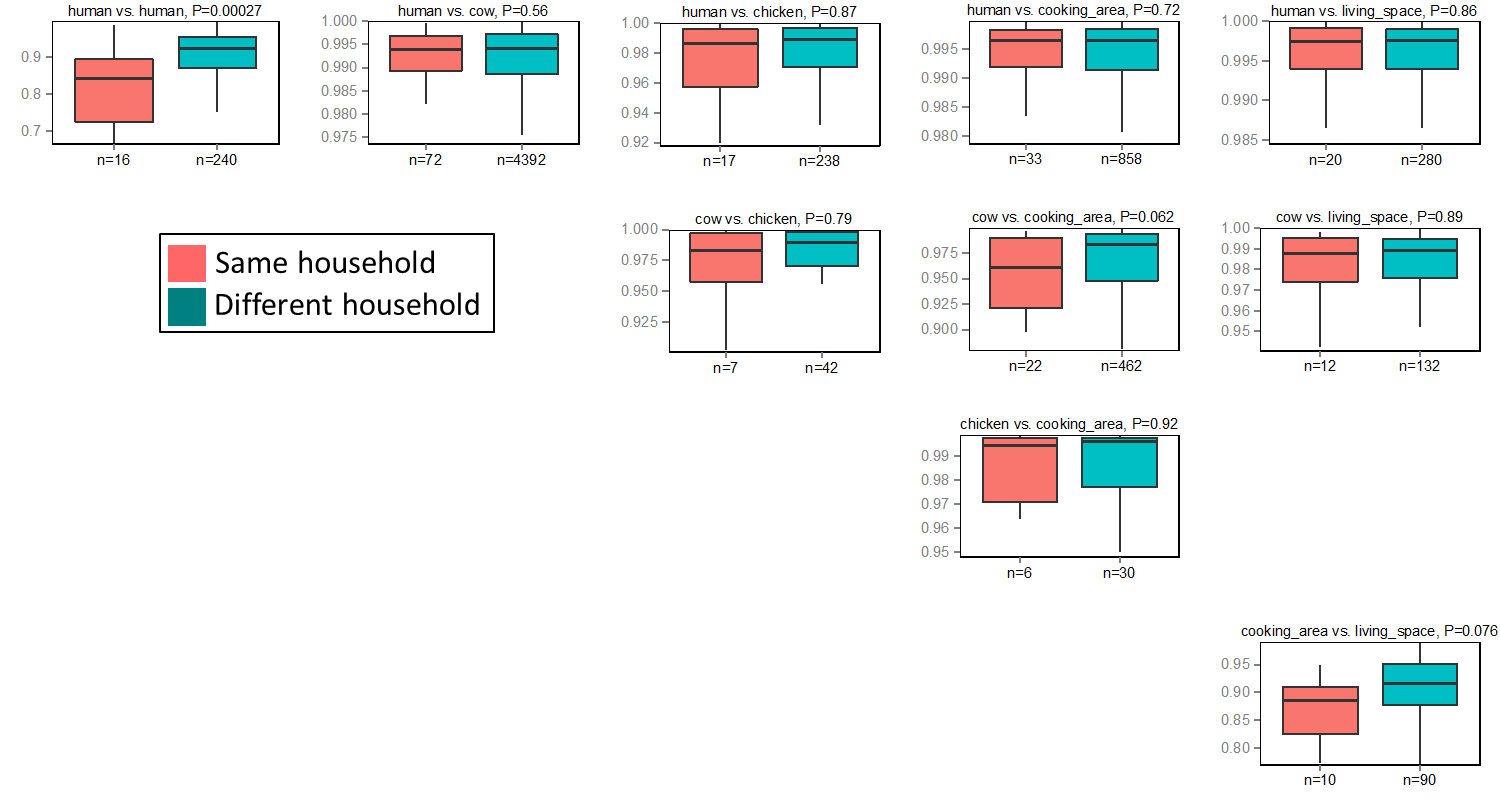

Supplement: S3 Fig — (TIF) [file pone.0171017.s003.tif]

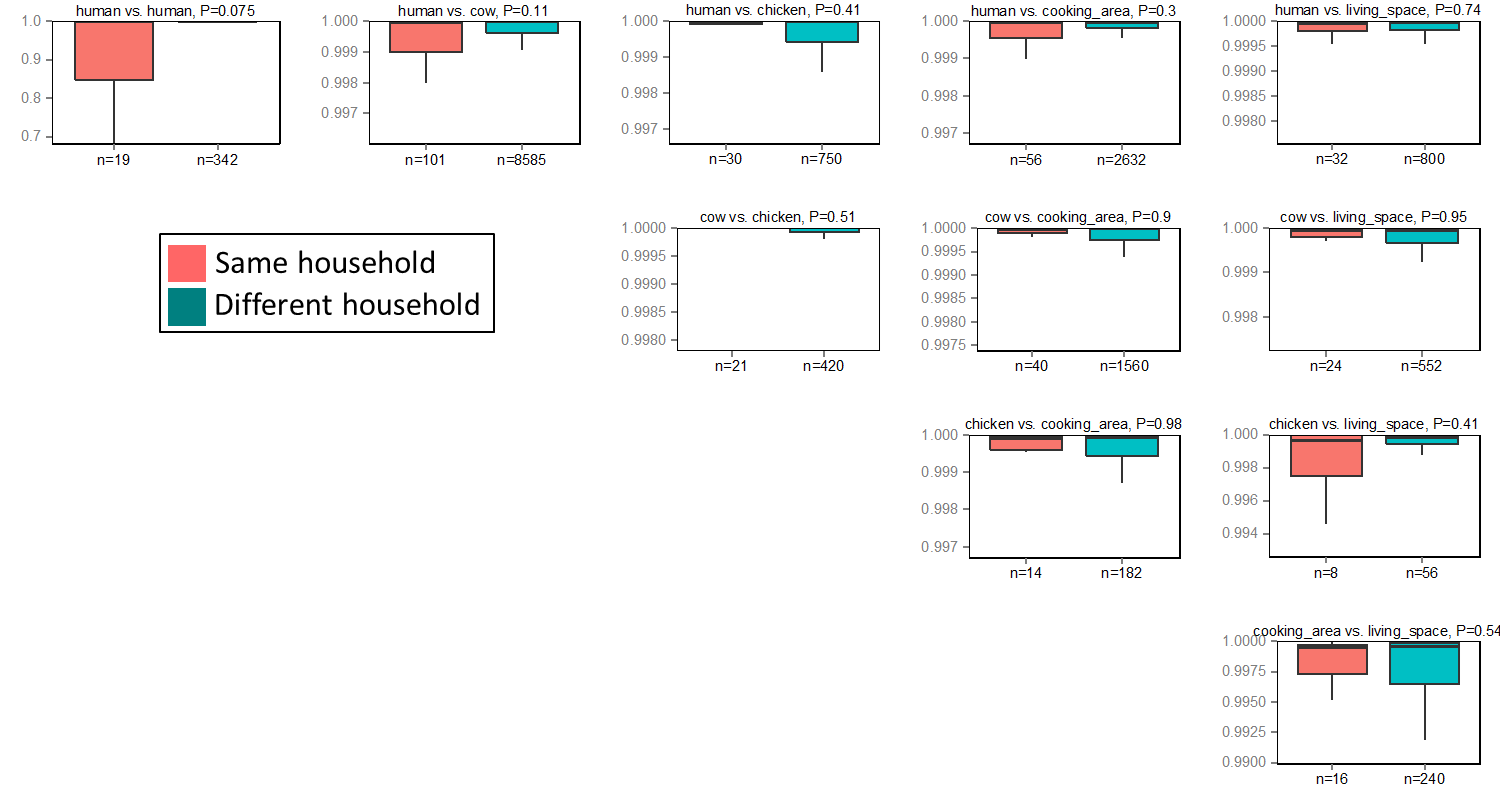

Supplement: S4 Fig — (TIF) [file pone.0171017.s004.tif]

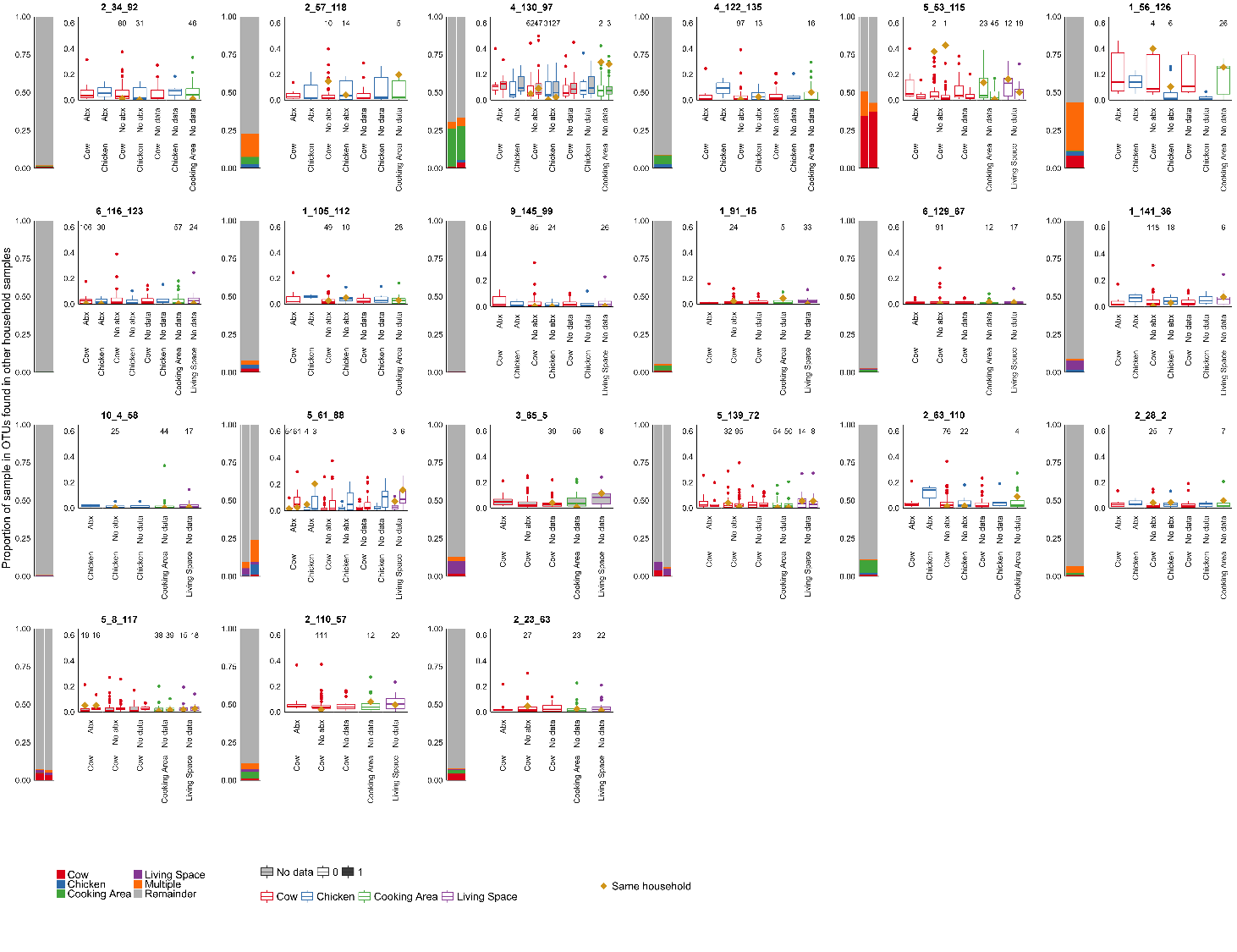

Supplement: S5 Fig — Each household is represented by on panel including the vertical bar and box plots. The bar graphs on the right side of each panel show the proportion of microbes shared between the child and their cow, chicken, cooking area, or living space. The box plots in each panel show the distribution of microbiome sharing comparing the child to samples from other households, compared to the proportion shared with their own household sample, represented by a gold diamond. (TIF) [file pone.0171017.s005.tif]

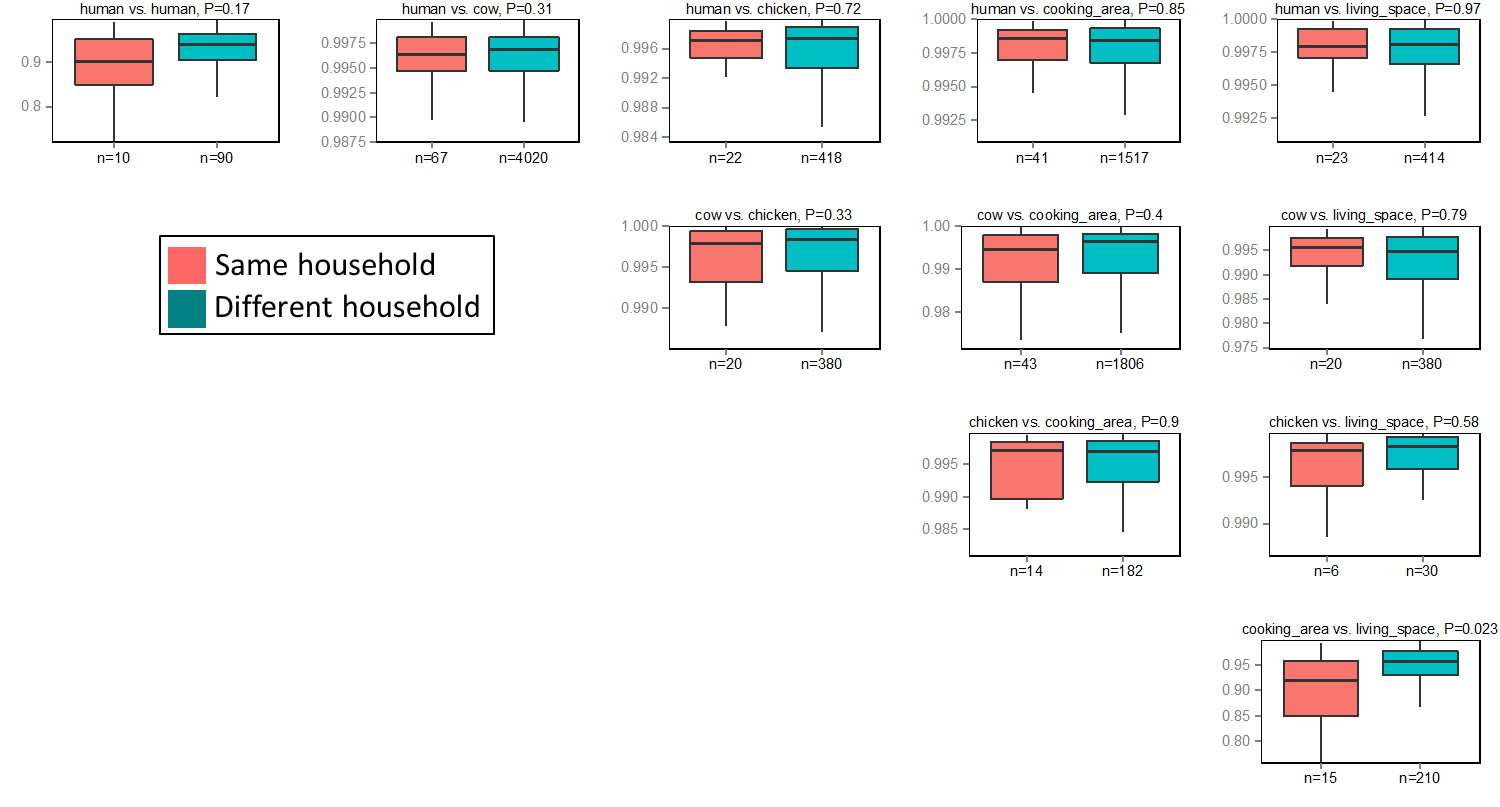

Supplement: S6 Fig — (TIF) [file pone.0171017.s006.tif]
