# Supplementary material for: Microbiome sharing between children, livestock and household surfaces in western Kenya
Source: PLoS One. 2017 Feb 2;12(2):e0171017. doi: 10.1371/journal.pone.0171017 (PMC5289499; doi:10.1371/journal.pone.0171017)
Supplement: S1 Table — (DOCX) [file pone.0171017.s007.docx]

**S1 Table**: Primer designs for PCR steps 1 and 2

| Primer name | PCR step | Oligo sequence 5’ 🡪 3’ |
| --- | --- | --- |
| CS1_8F_X | PCR-1 for | **ACACTGACGACATGGTTCTACA**(N_n_)*GTAGAGTTTGATCATGGCTCAG* |
| CS2_517R_X | PCR-1 rev | **TACGGTAGCAGAGACTTGGTCT**(N_n_)*CCATTACCGCGGCTGCTGG* |
| P5-leela_X_CS1 | PCR-2 for | AATGATACGGCGACCACCGAGATCTACAC(NNNNNNNN)**ACACTGACGACATGGTTCTACA** |
| P7-leela_X_CS2 | PCR-2 rev | CAAGCAGAAGACGGCATACGAGAT(NNNNNNNN)**TACGGTAGCAGAGACTTGGTCT** |
