# Supplementary material for: Microbiome sharing between children, livestock and household surfaces in western Kenya
Source: PLoS One. 2017 Feb 2;12(2):e0171017. doi: 10.1371/journal.pone.0171017 (PMC5289499; doi:10.1371/journal.pone.0171017)
Supplement: S2 Table — (DOCX) [file pone.0171017.s008.docx]

**S2 Table**: Master primer table with exact primer sequences

| Primer name | Oligo sequence (5’🡪3’) |
| --- | --- |
| CS1_8F_1 | ACACTGACGACATGGTTCTACAGTAGAGTTTGATCCTGGCTCAG |
| CS1_8F_2 | ACACTGACGACATGGTTCTACACGTAGAGTTTGATCATGGCTCAG |
| CS1_8F_3 | ACACTGACGACATGGTTCTACAACGTAGAGTTTGATTCTGGCTCAG |
| CS1_8F_4 | ACACTGACGACATGGTTCTACATACGTAGAGTTTGATTATGGCTCAG |
| CS1_8F_5 | ACACTGACGACATGGTTCTACAGTACGTAGGGTTCGATTCTGGCTCAG |
| CS1_8F_6 | ACACTGACGACATGGTTCTACACGTACGTAGAGTTTGATCCTGGCTTAG |
| CS1_8F_7 | ACACTGACGACATGGTTCTACAACGTACGTAGAATTTGATCTTGGTTCAG |
| CS2_517R_1 | TACGGTAGCAGAGACTTGGTCTCCATTACCGCGGCTGCTGG |
| CS2_517R_2 | TACGGTAGCAGAGACTTGGTCTGCCATTACCGCGGCTGCTGG |
| CS2_517R_3 | TACGGTAGCAGAGACTTGGTCTTGCCATTACCGCGGCTGCTGG |
| CS2_517R_4 | TACGGTAGCAGAGACTTGGTCTATGCCATTACCGCGGCTGCTGG |
| CS2_517R_5 | TACGGTAGCAGAGACTTGGTCTCATGCCATTACCGCGGCTGCTGG |
| P5-Leela_1-CS1 | AATGATACGGCGACCACCGAGATCTACACTAGATCGCACACTGACGACATGGTTCTACA |
| P5-Leela_2-CS1 | AATGATACGGCGACCACCGAGATCTACACCTCTCTATACACTGACGACATGGTTCTACA |
| P5-Leela_3-CS1 | AATGATACGGCGACCACCGAGATCTACACTATCCTCTACACTGACGACATGGTTCTACA |
| P5-Leela_4-CS1 | AATGATACGGCGACCACCGAGATCTACACAGAGTAGAACACTGACGACATGGTTCTACA |
| P5-Leela_5-CS1 | AATGATACGGCGACCACCGAGATCTACACGTAAGGAGACACTGACGACATGGTTCTACA |
| P5-Leela_6-CS1 | AATGATACGGCGACCACCGAGATCTACACACTGCATAACACTGACGACATGGTTCTACA |
| P5-Leela_7-CS1 | AATGATACGGCGACCACCGAGATCTACACAAGGAGTAACACTGACGACATGGTTCTACA |
| P5-Leela_8-CS1 | AATGATACGGCGACCACCGAGATCTACACCTAAGCCTACACTGACGACATGGTTCTACA |
| P5-Leela_9-CS1 | AATGATACGGCGACCACCGAGATCTACACTGAACCTTACACTGACGACATGGTTCTACA |
| P5-Leela_10-CS1 | AATGATACGGCGACCACCGAGATCTACACTGCTAAGTACACTGACGACATGGTTCTACA |
| P5-Leela_11-CS1 | AATGATACGGCGACCACCGAGATCTACACTGTTCTCTACACTGACGACATGGTTCTACA |
| P5-Leela_12-CS1 | AATGATACGGCGACCACCGAGATCTACACTAAGACACACACTGACGACATGGTTCTACA |
| P5-Leela_13-CS1 | AATGATACGGCGACCACCGAGATCTACACCTAATCGAACACTGACGACATGGTTCTACA |
| P5-Leela_14-CS1 | AATGATACGGCGACCACCGAGATCTACACCTAGAACAACACTGACGACATGGTTCTACA |
| P5-Leela_15-CS1 | AATGATACGGCGACCACCGAGATCTACACTAAGTTCCACACTGACGACATGGTTCTACA |
| P5-Leela_16-CS1 | AATGATACGGCGACCACCGAGATCTACACTAGACCTAACACTGACGACATGGTTCTACA |
| P5-Leela_17-CS1 | AATGATACGGCGACCACCGAGATCTACACTATAGCCTACACTGACGACATGGTTCTACA |
| P5-Leela_18-CS1 | AATGATACGGCGACCACCGAGATCTACACATAGAGGCACACTGACGACATGGTTCTACA |
| P5-Leela_19-CS1 | AATGATACGGCGACCACCGAGATCTACACCCTATCCTACACTGACGACATGGTTCTACA |
| P5-Leela_20-CS1 | AATGATACGGCGACCACCGAGATCTACACGGCTCTGAACACTGACGACATGGTTCTACA |
| P5-Leela_21-CS1 | AATGATACGGCGACCACCGAGATCTACACAGGCGAAGACACTGACGACATGGTTCTACA |
| P5-Leela_22-CS1 | AATGATACGGCGACCACCGAGATCTACACTAATCTTAACACTGACGACATGGTTCTACA |
| P5-Leela_23-CS1 | AATGATACGGCGACCACCGAGATCTACACCAGGACGTACACTGACGACATGGTTCTACA |
| P5-Leela_24-CS1 | AATGATACGGCGACCACCGAGATCTACACGTACTGACACACTGACGACATGGTTCTACA |
| P7-Leela_1-CS2 | CAAGCAGAAGACGGCATACGAGATTAAGGCGATACGGTAGCAGAGACTTGGTCT |
| P7-Leela_2-CS2 | CAAGCAGAAGACGGCATACGAGATCGTACTAGTACGGTAGCAGAGACTTGGTCT |
| P7-Leela_3-CS2 | CAAGCAGAAGACGGCATACGAGATAGGCAGAATACGGTAGCAGAGACTTGGTCT |
| P7-Leela_4-CS2 | CAAGCAGAAGACGGCATACGAGATTCCTGAGCTACGGTAGCAGAGACTTGGTCT |
| P7-Leela_5-CS2 | CAAGCAGAAGACGGCATACGAGATGGACTCCTTACGGTAGCAGAGACTTGGTCT |
| P7-Leela_6-CS2 | CAAGCAGAAGACGGCATACGAGATTAGGCATGTACGGTAGCAGAGACTTGGTCT |
| P7-Leela_7-CS2 | CAAGCAGAAGACGGCATACGAGATCTCTCTACTACGGTAGCAGAGACTTGGTCT |
| P7-Leela_8-CS2 | CAAGCAGAAGACGGCATACGAGATCAGAGAGGTACGGTAGCAGAGACTTGGTCT |
| P7-Leela_9-CS2 | CAAGCAGAAGACGGCATACGAGATGCTACGCTTACGGTAGCAGAGACTTGGTCT |
| P7-Leela_10-CS2 | CAAGCAGAAGACGGCATACGAGATCGAGGCTGTACGGTAGCAGAGACTTGGTCT |
| P7-Leela_11-CS2 | CAAGCAGAAGACGGCATACGAGATAAGAGGCATACGGTAGCAGAGACTTGGTCT |
| P7-Leela_12-CS2 | CAAGCAGAAGACGGCATACGAGATGTAGAGGATACGGTAGCAGAGACTTGGTCT |
| P7-Leela_13-CS2 | CAAGCAGAAGACGGCATACGAGATATCACGACTACGGTAGCAGAGACTTGGTCT |
| P7-Leela_14-CS2 | CAAGCAGAAGACGGCATACGAGATACAGTGGTTACGGTAGCAGAGACTTGGTCT |
| P7-Leela_15-CS2 | CAAGCAGAAGACGGCATACGAGATCAGATCCATACGGTAGCAGAGACTTGGTCT |
| P7-Leela_16-CS2 | CAAGCAGAAGACGGCATACGAGATACAAACGGTACGGTAGCAGAGACTTGGTCT |
| P7-Leela_17-CS2 | CAAGCAGAAGACGGCATACGAGATACCCAGCATACGGTAGCAGAGACTTGGTCT |
| P7-Leela_18-CS2 | CAAGCAGAAGACGGCATACGAGATAACCCCTCTACGGTAGCAGAGACTTGGTCT |
| P7-Leela_19-CS2 | CAAGCAGAAGACGGCATACGAGATCCCAACCTTACGGTAGCAGAGACTTGGTCT |
| P7-Leela_20-CS2 | CAAGCAGAAGACGGCATACGAGATCACCACACTACGGTAGCAGAGACTTGGTCT |
| P7-Leela_21-CS2 | CAAGCAGAAGACGGCATACGAGATGAAACCCATACGGTAGCAGAGACTTGGTCT |
| P7-Leela_22-CS2 | CAAGCAGAAGACGGCATACGAGATTGTGACCATACGGTAGCAGAGACTTGGTCT |
| P7-Leela_23-CS2 | CAAGCAGAAGACGGCATACGAGATAGGGTCAATACGGTAGCAGAGACTTGGTCT |
| P7-Leela_24-CS2 | CAAGCAGAAGACGGCATACGAGATAGGAGTGGTACGGTAGCAGAGACTTGGTCT |
| P7-Leela_25-CS2 | CAAGCAGAAGACGGCATACGAGATATTACTCGTACGGTAGCAGAGACTTGGTCT |
| P7-Leela_26-CS2 | CAAGCAGAAGACGGCATACGAGATTCCGGAGATACGGTAGCAGAGACTTGGTCT |
| P7-Leela_27-CS2 | CAAGCAGAAGACGGCATACGAGATCGCTCATTTACGGTAGCAGAGACTTGGTCT |
| P7-Leela_28-CS2 | CAAGCAGAAGACGGCATACGAGATGAGATTCCTACGGTAGCAGAGACTTGGTCT |
| P7-Leela_29-CS2 | CAAGCAGAAGACGGCATACGAGATATTCAGAATACGGTAGCAGAGACTTGGTCT |
| P7-Leela_30-CS2 | CAAGCAGAAGACGGCATACGAGATGAATTCGTTACGGTAGCAGAGACTTGGTCT |
| P7-Leela_31-CS2 | CAAGCAGAAGACGGCATACGAGATCTGAAGCTTACGGTAGCAGAGACTTGGTCT |
| P7-Leela_32-CS2 | CAAGCAGAAGACGGCATACGAGATTAATGCGCTACGGTAGCAGAGACTTGGTCT |
| P7-Leela_33-CS2 | CAAGCAGAAGACGGCATACGAGATCGGCTATGTACGGTAGCAGAGACTTGGTCT |
| P7-Leela_34-CS2 | CAAGCAGAAGACGGCATACGAGATTCCGCGAATACGGTAGCAGAGACTTGGTCT |
| P7-Leela_35-CS2 | CAAGCAGAAGACGGCATACGAGATTCTCGCGCTACGGTAGCAGAGACTTGGTCT |
| P7-Leela_36-CS2 | CAAGCAGAAGACGGCATACGAGATAGCGATAGTACGGTAGCAGAGACTTGGTCT |
