# Supplementary material for: Microbiome sharing between children, livestock and household surfaces in western Kenya
Source: PLoS One. 2017 Feb 2;12(2):e0171017. doi: 10.1371/journal.pone.0171017 (PMC5289499; doi:10.1371/journal.pone.0171017)
Supplement: S4 Table — (DOCX) [file pone.0171017.s010.docx]

**S4 Table**: Correlates of individual-level diversity (rarefied OTU count), multivariable linear mixed regression (N=107)

| Characteristic | Unadjusted Estimate (95% CI) | Adjusted Estimate (95% CI) |
| --- | --- | --- |
| Child age, months | 6.4 (1.8, 11.1) | 3.9 (-0.95, 8.7) |
| Child received antibiotics** | -84.4 (-220, 51.3) | -104.8 (-245.5, 35.8) |
| Child feeds livestock, yes/no | 177.6 (45.0, 310.2) | 164.0 (27.8, 300.3) |
| Household wealth*, score | -46.7 (-87.3, -6.2) | -57.4 (-100.2, -14.6) |

*Mean asset-based principal component score over 9 months

**In the prior one month before sample collection
