# Supplementary material for: Microbiome sharing between children, livestock and household surfaces in western Kenya
Source: PLoS One. 2017 Feb 2;12(2):e0171017. doi: 10.1371/journal.pone.0171017 (PMC5289499; doi:10.1371/journal.pone.0171017)
Supplement: S5 Table — (DOCX) [file pone.0171017.s011.docx]

**S5 Table**: Correlates of microbial similarity (Bray-Curtis distance metric) between children (N=22)

| Characteristic | Unadjusted* Estimate† (95% CI) |
| --- | --- |
| Total household livestock owned, count | -0.006 (-0.009, -0.002) |
| Number of household latrines | 0.06 (-0.002, 0.11) |
| Antibiotics** (ref=neither child) |  |
| One of two children received antibiotics | -0.04 (-0.10, 0.02) |
| Both children received antibiotics | -0.20 (-0.29, -0.11) |

*Multivariable analysis was not conducted due to low numbers

**In the prior one month before sample collection

†Negative estimates represent closer similarity in microbial communities. Positive estimates represent more divergence in microbial communities.
