# Supplementary material for: Microbiome sharing between children, livestock and household surfaces in western Kenya
Source: PLoS One. 2017 Feb 2;12(2):e0171017. doi: 10.1371/journal.pone.0171017 (PMC5289499; doi:10.1371/journal.pone.0171017)
Supplement: S1 File — (DOCX) [file pone.0171017.s012.docx]

**SUPPLEMENTAL MATERIALS**

**Supplementary material contents:**

Supplemental Methods:

- Sample collection
- PCR conditions
- Primer sequences – S1 and S2 Tables
- OTU picking methods

Supplemental Results:

- Table of individual-level microbiome data
- Sharing comparison: Jaccard binary distance metric with de novo OTU picking
- Sharing comparison: Bray Curtis plots closed OTU picking
- Sharing comparison: Bray Curtis plots Swarm OTU picking
- Multivariable correlates of individual diversity
- Multivariable correlates of household sharing

**SUPPLEMENTAL METHODS**

**Sample collection:** Sample collection was conducted over two days for each household. On the first day, community interviewers explained the purpose of the study and requested consent for enrollment from the head of household. After the informed consent was given, the community interviewers asked survey questions regarding the particular child’s contact with animals, animal sleeping arrangements, and antibiotic use among the children and animals. The team then provided a stool container and detailed instructions for the caregiver to collect the first available stool from the enrolled children the next morning. The community interviewers instructed the child’s caregiver to, using the provided gloves, aseptically collect at least 1ml of the child’s stool onto a piece of provided paper, or to use a diaper for young children, and then scrape the stool into the stool container using the sterile scoop attached to the inside lid of the sterile collection cups. The caregivers were requested to store the stool cup in a provided sealed Ziploc bag in a cool area out of direct sunlight and call the study toll-free line right away. The household head was also requested to keep one selected cow and one selected chicken at home the next day for the animal fecal sampling.

The caregiver was given a toll-free line to call the next morning as soon as the child’s sample was available. Previous work has shown that holding samples at room-temperature for up to three days has been shown not to significantly impact the diversity of the fecal microbiome giving us confidence that our rapid collection from the field gave us representative samples [1]. The team of two veterinary technicians visited the household within one hour of the call to collect the child’s stool sample and the animal fecal samples. The team explained the criteria for contamination to the caregiver and asked if the sample had been contaminated or left in the sun. If the team perceived that there might be any contamination, delay in access to the sample, or insufficient sample, they requested a new stool sample to pick up the next day.

After deeming the child’s stool sample to have been collected cleanly, the team placed the sample into an ice box for transportation. The veterinary technicians then proceeded to collect cow, chicken, and household environmental samples. All animal samples were collected in alignment with animal surveillance system care guidelines using sterile materials and appropriate personal protective equipment. To minimize stress and risk of injury, the chickens were wrapped in a light towel with their eyes covered. The veterinary technicians held the chickens securely against the chest of the restrainer for no more than 2 minutes, while a second technician gently swabbed the cloaca with a sterile swab. Swabs were placed in a sterile Whirl-Pak® without transport medium and the bag was placed in a cooler for transportation. Cows were restrained using a head halter for less than 5 minutes in order to obtain a fecal sample directly from the rectum through digital manipulation. Cattle fecal samples were collected into a stool container and placed in a cool box with ice for transportation. The team also collected environmental swabs from two locations: food preparation area and the main floor of the living area. To conduct this sampling, the team soaked a sterile 2-in x 2-in piece of gauze in 1-mL of sterile ddH_2_O. The technicians swabbed a 10cm x 10cm area with the moistened gauze pad and placed the pad into a sterile Whirl-Pak bag. All samples were given unique individual sample barcodes to accurately track samples and were individually wrapped in Whirl-Pak® bags to prevent cross-contamination.

Samples were not accepted in the laboratory if they were obviously contaminated with other material (e.g. feather in with cloaca swab, dirt in fecal sample, sticks in fecal sample), the sample ID numbers did not align with sample sheets, packaging was not sufficient to prevent cross contamination between samples, the tracking forms indicate contamination was reported by the caregiver, the sample was not stored properly in field, or the sample arrived to lab without maintaining cool temperature.

**PCR amplification details:**

PCR conditions were as follows: PCR-1 consisted of a 50 µL reaction containing 1X PCR Buffer (New England Biolabs), 3 mM MgCl_2_ (New England Biolabs), 0.24 mg/mL BSA (Fermentas), 200 nM dNTP mix (Fermentas), 50 nM of each Forward and Reverse primer, 0.025 U/uL of Taq DNA Polymerase (New England Biolabs) and up to 10 ng of template DNA. Cycling conditions for PCR-1 were 95˚C for 2 min, followed by 20 cycles of (95˚C – 1 min, 51˚C – 1 min, 72˚C – 1 min), a final extension of 72˚C – 10 min, and finally a 10˚C hold. PCR-2 consisted of a 20 µL reaction volume containing 1X PCR buffer, 4.5 mM MgCl_2_, 0.24 mg/mL BSA, 200 nM dNTPs, 75 nM each Forward and Reverse primers, 0.05 U/µL Taq DNA Polymerase and 1 µL of diluted PCR-1 product. Cycling conditions for PCR-2 were 95˚C for 10 min, followed by 10 cycles of (95˚C – 15 sec, 51˚C – 30 sec, 72˚C – 1min), a final extension at 72˚C – 3, and finally a 10˚C hold.

In primers CS1_8F_X and CS2_517R_X *Italicized* sequences represent the 008F and 517R ‘universal’ 16s-rRNA primers, respectively which match the target sequence. The (N_n_) sequence represents additional nucleotides added to the 5’ end of the gene specific portion of the oligo (between the CS1 or CS2 tag and core primer sequence) for the purpose of increasing diversity in the first 5-6 cycles of the sequencing run during cluster identification and phasing/prephasing color adjustments. **Bold** sequences represent the CS1 and CS2 tags.

In primers P5-leela_X_CS1 and P7-leela_X_CS2 the underlined sequences represent the P5 and P7 sequencing adaptors, respectively, used by Illumina sequencing to bind the molecules to the flow cell. The 8 nucleotide sequence (NNNNNNNN) is a specific barcode ID for multiplexing samples. **Bold** sequences again represent the CS1 and CS2 tags so that PCR-2 primers are able to amplify off of amplicons generated during PCR-1.

The “X” in the primer names correspond to number assigned to that particular barcode utilized during de-multiplexing of sequences. All exact primer sequences used are given in supplementary S2 Table.

**OTU Picking Methods:**

**Closed reference OTU picking**

To compare abundances of known microbial taxa, we used closed reference OTU picking against the 16S rRNA portion of the SILVA database, version 1.2.3, formatted for QIIME by Tony Walters and the QIIME development team [2, 3]. OTUs with fewer than two reads (singletons) were filtered out. The resulting OTU table was rarefied to the number of reads of the sample with the fewest reads greater than 2500 (2536). OTUs present in only one sample were also filtered. For taxonomic summaries, we used the SILVA majority 7-level OTU taxonomic assignments.

We compared the results of closed-reference OTU picking between the larger and more recently updated SILVA database with the QIIME default Greengenes database. 15.6% more reads were assigned reference OTUs using SILVA than Greengenes (6,034,219 versus 5,219,741). Only 4,452,496 reads have assignments from both databases. Of these, 91.8% were assigned to the same phylum, 83.9% were assigned to the same class, and 77.6% were assigned to the same order. We chose to use the results from the SILVA database for further analyses.

***De novo* OTU picking**

*De novo* OTU picking was performed using uclust in QIIME. The uclust *maxrejects* parameter was set to 24 to improve cluster quality. Representative sequences for each OTU were assigned taxonomy based on similarity to SILVA 1.2.3 97% reference 16S OTUs using uclust. Singleton OTUs were again filtered. We used PyNAST to align representative sequences against the SILVA 1.2.3 16S gene core alignment, and OTUs with less than 65% identity to any sequence in the reference were filtered out. Representative sequences were also checked for chimeras using the uchime-ref algorithm implemented in *vsearch*, and OTUs identified as chimeric were removed [2]. Lastly, a phylogenetic tree of representative sequences was constructed based on the filtered alignment using FastTree [4].

**Swarm OTU clustering**

Swarm v2 [5] uses iterative single-linkage nearest-neighbor clustering to group sequences into OTUs. We applied Swarm clustering with a local clustering threshold of *d*=1 to characterize higher-resolution strain diversity. We followed the recommended Swarm usage, de-replicating sequences using *vsearch*, clustering reads, and then applying the same chimera checking, taxonomy assignment, singleton filtering, alignment, and phylogenetic tree construction pipeline as for uclust *de novo* clustering.

**Supplemental Materials References:**

1. Dominianni CJ, Wu J, Hayes RB, and Ahn J: **Comparison of methods for fecal microbiome biospecimen collection.** *BMC Microbiology* 2014, **14**: 103.
2. Edgar RC: **Search and clustering orders of magnitude faster than BLAST**. *Bioinformatics* 2010, **26**(19): 2460-2461.
3. Quast CE, Pruesse E, Yilmaz P, Gerken J, Schweer T, Yarza P, Peplies J, Glöckner FO: **The SILVA ribosomal RNA gene database project: improved data processing and web-based tools**. *Nucleic Acids Research* 2013, **41**(Database issue): D590-596.
4. Price MN, Dehal PS, and Arkin AP: **FastTree: computing large minimum evolution trees with profiles instead of a distance matrix**. *Molecular Biology and Evolution* 2009, **26**(7): 1641-1650.
5. Mahé F, Rognes T, Quince C, de Vargas C, and Dunthorn M: **Swarm v2: highly-scalable and high-resolution amplicon clustering** *PeerJ* 2015, **3**: e1420.
